# Juvenile confinement exacerbates adversity burden: A neurobiological impetus for decarceration

**DOI:** 10.3389/fnins.2022.1004335

**Published:** 2022-09-30

**Authors:** Natalia Orendain, Adriana Galván, Emma Smith, Elizabeth S. Barnert, Paul J. Chung

**Affiliations:** ^1^Department of Psychiatry and Biobehavioral Sciences, University of California, Los Angeles, Los Angeles, CA, United States; ^2^David Geffen School of Medicine, Semel Institute for Neuroscience and Human Behavior, University of California, Los Angeles, Los Angeles, CA, United States; ^3^Department of Psychology, University of California, Los Angeles, Los Angeles, CA, United States; ^4^Department of Pediatrics, David Geffen School of Medicine, University of California, Los Angeles, Los Angeles, CA, United States; ^5^Department of Health Systems Science, Kaiser Permanente Bernard J. Tyson School of Medicine, Pasadena, CA, United States

**Keywords:** neurodevelopment, adversity, trauma, stress, juvenile justice, adolescence, incarceration

## Abstract

Every year, about 700,000 youth arrests occur in the United States, creating significant neurodevelopmental strain; this is especially concerning as most of these youth have early life adversity exposures that may alter brain development. Males, Black, and Latinx youth, and individuals from low socioeconomic status households have disproportionate contact with the juvenile justice system (JJS). Youth confined in the JJS are frequently exposed to threat and abuse, in addition to separation from family and other social supports. Youths’ educational and exploratory behaviors and activities are substantially restricted, and youth are confined to sterile environments that often lack sufficient enrichment resources. In addition to their demonstrated ineffectiveness in preventing future delinquent behaviors, high recidivism rates, and costs, juvenile conditions of confinement likely exacerbate youths’ adversity burden and neurodevelopmentally harm youth during the temporally sensitive window of adolescence. Developmentally appropriate methods that capitalize on adolescents’ unique rehabilitative potential should be instated through interventions that minimize confinement. Such changes would require joint advocacy from the pediatric and behavioral health care communities. “The distinct nature of children, their initial dependent, and developmental state, their unique human potential as well as their vulnerability, all demand the need for more, rather than less, legal and other protection from all forms of violence (United Nations Committee on the Rights of the Child, 2007).”

## Introduction

When the United Nation’s minimum age of legal culpability, competence to stand trial, and capacity to intentionally perform wrongful behavior was defined, neurodevelopmental research was nascent. Now, neurodevelopmental research is taking an essential seat not only in the discussion of culpability, but in the treatment of delinquent behavior. In our discussion, we—a team of neuroscientists and pediatricians—advise that the current conditions of juvenile confinement can serve as a form of adversity, which can further contribute to negative neurodevelopmental and quality of life outcomes. Adversity is defined as exposure to abuse, neglect, and pervasive threat; exposure occurring before the age of 18 years is termed early life adversity. An individual’s adversity burden is their cumulative lifetime exposure. Oftentimes, terms such as chronic stress and trauma are used interchangeably with adversity exposure.

The United States’ Juvenile Justice System (JJS) derives much of its structure from the adult criminal justice system and operates in a similarly punitive manner (McCarthy et al., 2016), with confinement serving as both incapacitation and deterrence from crime. Males, racial and ethnic minorities, and youth from socioeconomically disadvantaged households are disproportionately impacted (Spinney et al., 2018; Padgaonkar et al., 2020).

As the majority of JJS-involved youth enter the system with prior adversity exposure, experiencing adversity while confined can further exacerbate the detrimental neurodevelopmental outcomes associated with such exposures (Lansing et al., 2016), creating a cumulative disadvantage (Chapman et al., 2006; Logan-Greene et al., 2016). Developmental science suggests that the current JJS is often developmentally inappropriate and may culminate in additional adversity burden for some of our most disadvantaged and vulnerable youth.

## Defining the reach of the justice system on young children

The United Nations (UN) Convention on the Rights of the Child of 1989 defined any and every human being below the age of eighteen a *child*—unless majority is attained earlier under law. In 2019, the UN Committee on the Rights of the Child recommended fourteen as the absolute minimum age at which children can be held legally responsible for their actions, raising the international standard for a minimum age of juvenile confinement from twelve. The United States (US) is the only country to not have ratified the Convention, and most US states have not adopted a minimum age of legal responsibility. Among states that have, fourteen states have established 10 years as the minimum age, and North Carolina has set it at six.

## The juvenile carceral continuum

The US criminal justice system incarcerates more individuals per capita—including its youth—than any other country in the world. Despite declining arrest rates, about 700,000 youth arrests still occur every year, with the most common offenses being: larceny-theft, drug abuse violations, disorderly conduct, and vandalism (Lansing et al., 2016; Office of Juvenile Justice and Delinquency Juvenile Arrests, 2021). Youth arrest rates for violent crimes (e.g., robbery and assault) comprise only about 6% of all juvenile arrests and are near their lowest in over 40 years (Office of Juvenile Justice and Delinquency Juvenile Arrests, 2021). Aggression, conduct problems, and psychopathy typically desist with age (Bos et al., 2018), with 90% of juvenile offenders desisting from crime by their mid-20’s (Steinberg, 2013).

Racial disparities in incarceration rates are evident, particularly with Black (40%) and Latino (23%) male youth disproportionately represented (Barnert et al., 2019). Youth with cognitive, physical, and mental disabilities and youth who identify as lesbian, gay, bisexual, transgender, and questioning (LGBTQ) (20%) are also disproportionately involved with the justice system (Society for Adolescent Health and Medicine, 2016). Among JJS-involved youth, two-thirds of males and three-quarters of females present with at least one psychiatric diagnosis, such as substance abuse, depression, or post-traumatic stress disorder (PTSD) (Barnert et al., 2016).

Youth held in the JJS are confined to shared living quarters or isolation; receive limited and supervised contact with sources of support outside the facility; and often have minimal access to enrichment and educational opportunities (Mendel, 2015; McCarthy et al., 2016). Confined youth are also often without access to quality healthcare, sanitary living conditions, adequate sleep, and other basic needs (Barnert et al., 2016; Anderson et al., 2019). Furthermore, allegations of abuse (physical, sexual, and emotional) are routinely reported in the JJS and have induced staff relocations, resignations, and facility closures (Mendel, 2015; McCarthy et al., 2016). Psychosocial and neuroimaging research has demonstrated the detrimental impacts of abuse and neglect on adolescent neurodevelopment and behaviors (Teicher and Samson, 2016; McLaughlin et al., 2019) even in the presence of redeeming resources like education and life skills trainings (Fritz et al., 2018). Environmental onslaughts experienced during adolescents’ temporally sensitive window of neurodevelopment can leave a lifelong imprint on neural structure and functioning (Nettle et al., 2017). While variability exists among individual juvenile detention facilities, the JJS in practice often operates in a manner akin to the adult criminal justice system (Feld, 1997).

## Neurodevelopment

Sensitive windows of development are periods of both increased vulnerability to stressors and enhanced malleability in response to interventions, both of which can yield a lasting impact on brain structure and function (Lupien et al., 2009). Adolescence, defined roughly as the onset of puberty to the mid-20s, is one such window.

Contrary to childhood, which is characterized by global and regional neural outgrowth, adolescence is marked by refinement in neuronal processing, attributable to synaptic pruning that facilitates responding to anticipated environmental conditions. Declines in gray matter volume are attributed to neuronal firing efficiency, while white matter volumetric increases support whole brain connectivity and axonal myelination. The age of pubertal onset and the length of pubertal maturation influence the neurodevelopmental trajectory and behavior of youth (Blakemore et al., 2010; Herting and Sowell, 2017); however, neuroplasticity diminishes with age causing adolescent experiences to have a tremendous impact not only on this critical period but on adulthood.

**FIGURE 1:**
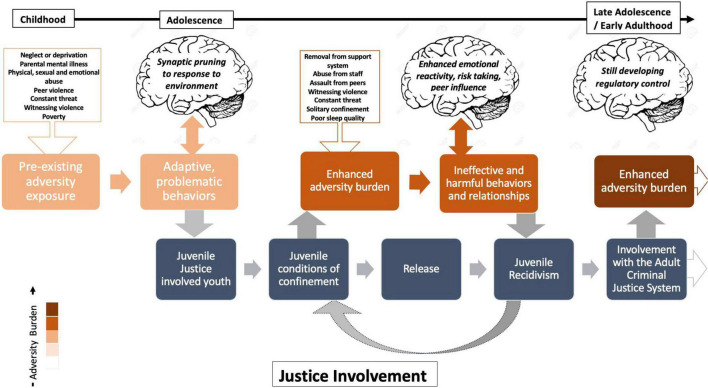
Conceptual representation of youth adversity exacerbation in the juvenile justice system (JJS).

Compared to other ages, the period of adolescence contains a disproportionate amount of both “offenders” and victims of crime (Society for Adolescent Health and Medicine, 2016). Adolescence is uniquely characterized by enhanced emotional reactivity and responding, increased impulsivity and risk taking, heightened peer influence, and continued maturation of regulatory control, in comparison with adults. Adversity exposure can amplify these normative adolescent behaviors (Lansing et al., 2016). Around 90% of JJ-involved youth have a history of adversity exposure (Baglivio and Epps, 2014), and one-third of JJ-involved youth have been exposed to five or more adverse childhood experiences (Barnert et al., 2019). Early life adversity refers to instances of abuse (physical, verbal, or sexual), neglect, or deprivation that occur during childhood and adolescence. Similar to neural refinement processes during adolescence that lead to stronger and more specialized connections, neural circuitry that is repeatedly engaged as a result of adversity exposure (Teicher and Samson, 2016; McLaughlin et al., 2019) is subsequently strengthened and therefore primed for recruitment with further onslaughts (Tottenham and Galván, 2016). Thus, youth with altered neurodevelopment due to adversity exposure are more vulnerable to the detrimental imprint of high stress environments, like detention facilities and prisons (Lansing et al., 2016; Barnert et al., 2019). We hypothesize that youth’s neurodevelopmental vulnerability to the conditions of confinement propels them toward trauma exacerbation and engagement in ineffective and harmful cognitive processing, behaviors and relationships. Engagement in these behaviors may yield future justice system involvement, as depicted in Figure 1.

## Stress and the brain

Prolonged and frequent activation of the body’s sympathetic nervous system [i.e., hypothalamic-pituitary-adrenal (HPA) axis] and circulation of stress hormones are detrimental to homeostatic maintenance and overall health (Lupien et al., 2009). Glucocorticoids permeate the blood-brain barrier and act on receptors in the frontal lobe, insula, amygdala, hippocampus, and cerebellum resulting in impaired functioning and structural alterations (Teicher and Samson, 2016). Neural regions impacted by stress typically contain a high concentration of glucocorticoid receptors, with early life adversity and stress hormone exposure resulting in decreased cell proliferation and neurogenesis, increased apoptosis, and diminished synaptic spine density (Bath et al., 2016). Given that the developing brain contains a greater proportion of stress hormone receptors than adults (Avishai-Eliner et al., 1996), youth are more vulnerable to the impacts of stress and adversity.

Extensive research has explored the impact of early life adversity on prominent neural circuitry, endocrine functioning, psychopathology, and behavior (Cowan et al., 2016; Teicher and Samson, 2016)—exposure akin to chronic stressors faced during juvenile confinement (Lansing et al., 2016). Different forms of early life adversity, such as abuse, neglect and deprivation—all present at a higher rate in JJS-involved youth—are suggested to uniquely impact the brain contingent upon the characteristics of the stressor, e.g., age of onset, duration, and frequency of exposure (Callaghan et al., 2014; Teicher and Samson, 2016). Physical abuse is the most widespread form of abuse early in life, particularly among males (Felitti et al., 1998; Teicher et al., 2012). Associated with a history of physical abuse is traumatic brain injury (TBI) (Ewing-Cobbs et al., 1999), the immediate effect being axonal injury resulting in cellular transport disruption, inflammation, and eventual white matter degeneration. Systematically, TBI negatively impacts cognitive control, intellectual function, attention, memory, social functioning, psychiatric symptomology, and is associated with delinquency (McKinlay, 2014). Youth are particularly vulnerable to the neural effects of TBI given the prevalence of developing fiber bundles and unmyelinated axons. A 2013 meta-analysis noted that juvenile offenders were over 3 times as likely to report a history of TBI than controls, with high rates among juvenile offenders both pre- and post-confinement (Farrer et al., 2013).

National survey data indicate that adolescent exposure to community violence is on par with adversity exposure within the home (Fagan et al., 2014). JJS-involved youth often present with a history of neighborhood violence exposure (Chapman et al., 2006) and originate from impoverished or low SES households (Wilson et al., 2009); these households are disproportionality Black and Latinx (Williams and Collins, 2001). Irrespective of direct harm, community violence exposure constitutes a pervasive threat that accelerates biological aging and contributes to detrimental quality of life outcomes (Motley et al., 2017; Sumner et al., 2019). Youth from low SES households have higher basal cortisol levels (Kuhlman et al., 2018), neural alterations similar to those associated with early life adversity exposure (Hanson et al., 2015) and present with advanced pubertal development. Many Black and Latinx youth undergo pubertal maturation earlier than White youth (Ramnitz and Lodish, 2013) and thus may outwardly physically appear older. Youth who appear more pubertally advanced are more often treated as adults and stigmatized in school and throughout the community (Mendle et al., 2019), despite their developing biology. This stigmatization based off outward appearances may in part explain prosecutorial attempts to erroneously place justice-involved youth in adult correctional facilities.

## Confinement as adversity

Instances of abuse—including physical, sexual and emotional—within the JJS are not uncommon. Evidence of youth maltreatment within the JJS has been documented in most states (Mendel, 2015), with maltreatment including violence, sexual abuse, and frequent or prolonged use of restraints. Punitive tactics such as physical force, group punishment, and solitary confinement are routinely employed at all levels of criminal justice involvement (Mendel, 2015; Owen and Wallace, 2020). Neuroscientific research on solitary confinement documents its damaging impact on mental and physical health, socioemotional learning, and overall quality of life. The acute and chronic repercussions of isolation include prominent behavioral and social deficits and a propensity for psychopathology, particularly mood disorders and psychosis; these effects have been observed across developmental periods and among numerous species, including rodents, non-human primates, adolescents, and adults (Champagne and Meaney, 2007; Sinclair et al., 2014). The most recent data indicate that over a third of youth in custody experienced solitary confinement (American Civil Liberties Union, 2013; Department of Justice, 2017). In 2016, the Obama administration banned the use of solitary confinement for juveniles held in federal prisons and tightened restrictions on its use for adult inmates. While the majority of youth are held under state jurisdiction, the federal isolation ban for juveniles has been a powerful catalyst for state and local reform.

It is well-established that enriched environments support optimal brain development through greater learning opportunities and enhanced neuroplasticity (Bailoo et al., 2018). The sterile environments youth are confined to in the JJS restrict behaviors and activities that are educational, exploratory, or creative (McCarthy et al., 2016). Despite supportive conditions in place—such as primary and secondary education, behavioral management, and skill building—the prevalence of physical violence, constant threat, absence of a stable support system, and seclusion is still detrimental to neurodevelopment and overall functioning (Mota et al., 2016). A proportional hazards model from an aggregated exposure of 386,709 person-years positively correlated the degree of justice involvement (i.e., arrest, detainment, confinement, or transfer to an adult facility) with mortality risk (i.e., homicide), such that youth transferred to an adult facility were more likely to die than detained youth (Aalsma et al., 2016). Evidence indicates that current conditions of juvenile confinement are developmentally harmful for confined youth and serve as a source of adversity exposure and possible exacerbation.

The COVID-19 pandemic has intensified the challenging living conditions and health risks faced by confined youth (Barnert, 2020). Online-learning resources available for confined youth are ill-equipped to meet their educational and rehabilitative needs; youth are further distanced from sources of support as visitations have been suspended or reduced (Barnert, 2020). The unique neurodevelopmental need of social support during adolescence is no less for these youth and of particular importance given the demonstrated impact of social support on decreasing the occurrence of problematic and delinquent behaviors (Fritz et al., 2018).

## Neuroscience applied to juvenile justice reform

Neurocognitive, psychosocial, and behavioral research has informed much of our understanding of criminal behavior, culpability, and the ensuing legal consequences. Recent neuroscientific research that underscores adolescents’ developmentally diminished culpability has been referenced in court cases and has impacted legal proceedings. The 2005 Supreme Court case of *Roper v. Simmons* eliminated the death penalty for individuals under age 18 by referencing scientific findings upholding the developmental immaturity of adolescents in terms of impulsivity, recklessness and irresponsibility, as well as heightened peer influence.

In 2010, the Supreme Court ruled in the case of *Graham v. Florida* that life sentences without the possibility of parole for juveniles convicted of non-homicidal crimes were cruel and unusual and thus unconstitutional. Building upon *Graham v. Florida*, the 2012 *Miller v. Alabama* Supreme Court case ruled *mandatory* sentencing of juvenile life without parole was in violation of the Eighth Amendment’s ban on cruel and unusual punishment. Youths’ inherent vulnerability to and dependence on their environment provided the impetus for the court’s decision.

Unlike the previous court cases predominately addressing youth sentencing, the 2011 Supreme Court case of *J.D.B. v. North Carolina* utilized neurodevelopmental research to inform our understanding of cognitive capacity. This case determined that age must be accounted for when conducting the *Miranda* police custody analysis. As youth’s cognitive faculties are still developing, their ability to reason, make long-term projections, and understand the legal gravity of any past actions and criminal involvement are not yet sufficiently within their developmental purview.

These Supreme Court decisions, along with other recent advancements in juvenile justice reform, bring the US in agreement with article 37(a) of the UN’s Convention on the Rights of the Child prohibiting capital punishment and life imprisonment without the possibility of release for individuals under eighteen. In each of these court cases, adolescents’ neurobiological propensity for risk taking, impulsivity and enhanced peer influence was weighed heavily. Examining neuroimaging scans across thousands of individuals was ultimately how the scientific community demonstrated that typical adolescent behaviors, which are conserved across species, can be attributed to unique, neurodevelopmentally appropriate characteristics of the adolescent brain. In addition to attention focused on youth’s characteristics pre-confinement, the long-term detrimental physical and mental health effects of juvenile confinement have been documented by health professionals (Barnert et al., 2019). Mitigating the detrimental impacts of juvenile confinement on neurodevelopment while improving health outcomes of previously incarcerated youth should provide the impetus for the next wave of advancements in JJS reform.

## Discussion

The US Department of Justice reports, “Nearly one in three Americans of working age have had an encounter with the criminal justice system” (Department of Justice, 2017). While an encounter encompasses any level of justice system involvement, the statistic highlights the system’s degree of enmeshment within our society. In both the juvenile and adult criminal justice systems, males and minoritized groups are overrepresented, even after controlling for type of crime, age, SES, and US geographic region (McCarthy et al., 2016). The annual fiscal burden placed on society from confining youth ranges from $8 to $21 billion (Justice Policy Institute, 2015). Additionally, juvenile recidivism rates are comparable to that of adults (Mendel, 2011) with 70–80% of youth facing re-arrest 2–3 years post-release (McCarthy et al., 2016). Adults with a history of juvenile confinement face significant disadvantages educationally, economically, socially, emotionally, interpersonally (Abram et al., 2017), and in general health (Barnert et al., 2016).

### Alternatives to the juvenile confinement model

Given that the current conditions can serve as a source of traumatization and adversity exacerbation, leaving youth more vulnerable to adverse outcomes than when they entered, scientists, clinicians, and advocates have called for the instatement of a more developmentally appropriate evidence-based continuum of services (Balsamo and Poncin, 2016; McCarthy et al., 2016; Lynch et al., 2018; Guckenburg et al., 2019). A 2009 meta-analysis of 548 studies on alternatives to confinement and youth recidivism found that interventions rooted in counseling, skill-building, and restoration had the greatest impact on recidivism reductions; conversely, programs built on deterrence and discipline were associated with higher reoffending rates (Lipsey, 2009). Thus, programs aimed at rehabilitation rather than punishment would capitalize upon adolescent neuroplasticity and be more effective at deterring adolescents from delinquent behaviors. Here, we provide a summary of evidence-based alternatives to youth confinement organized in three tiers based off youth’s history of involvement with the JJS and their current charge: (1) low-level, non-violent offenses; (2) non-violent offenses among individuals with a history of JJ involvement; and (3) violent, repeat offenses (Table 1).

**TABLE 1:** Alternatives to juvenile incarceration for all youth through the age of 25 years.

Youth characteristics	Proportion of youth	Rehabilitative resource	Example
Low-level, non-violent offenses	80–90%	Community-based diversion programs involving the family, including school-based, athletic associations, and peer mentorships	Arches transformative mentoring program
Non-violent offenses among individuals with a history of JJ involvement	10–15%	Monitored home placement incorporating evidence-based interventions, such as FFT, MST, and MTFC	Juvenile detention alternatives initiative
Violent, repeat offenses	<5%	Residential treatment and rehabilitation provided by social services, housed in a small facility within the community	Finland’s hybrid child welfare and juvenile justice system

JJ, juvenile justice; FFT, family therapy; MST, multisystemic therapy; MTFC, multidimensional treatment foster care.

The first-line intervention is culturally supportive and developmentally appropriate diversion tactics implemented within the community. These include school-based programs, athletic associations, and peer mentorships that involve the family (Farahmand et al., 2012). In New York City, the Department of Probation addressed neighborhood-specific challenges faced by youth and curbed recidivism through the Arches Transformative Mentoring Program. By pairing youth with paid mentors who are former inmates *and* from the same neighborhood of their mentees, program graduates committed significantly less felonies 1- and 2-years post-probation (Lynch et al., 2018). Environmental enrichment and social support have neuro-protective properties in their ability to diminish the detrimental structural and functional neurobiological correlates of stress while enhancing neuroplasticity (Biggio et al., 2019).

For youth unresponsive to diversion tactics within the community, the next line of intervention could be the home. Monitored home placement may be less disruptive to and more appropriate for developmental processes while youth are undergoing redirection and rehabilitation. Evidenced-based interventions include functional family therapy, multisystemic therapy, and multidimensional treatment foster care (Balsamo and Poncin, 2016). The Detention Diversion Advocacy Program and Juvenile Detention Alternatives Initiative follow this approach and have been recognized by governmental agencies as national models and evidence-based alternatives to juvenile confinement (The Annie E. Casey Foundation, 2017). Given adversity’s impact on frontolimbic circuitry, improving emotion regulation, particularly in females, in parallel with evidence-based interventions has been shown to improve treatment responsivity (Winiarski et al., 2017).

For the remaining youth for whom neither community diversion nor in-home rehabilitation is appropriate, community-based facilities staffed by a consortium of social service officers and health and educational professionals could be utilized (McCarthy et al., 2016; Society for Adolescent Health and Medicine, 2016). These facilities reflect an organizational shift from confinement in correctional facilities to residential treatment and rehabilitation provided by social services, as has historically been the case for youth in Finland (Abrams et al., 2018). In 2020, California bill SB823 shifted oversight of juvenile cases from state corrections to county-level health departments. The legislation aims to promote developmentally sensitive rehabilitation, house youth closer to sources of support, and permit youth to remain in the juvenile system until age 25, limiting transfers to adult corrections.

As reward sensitivity peaks during adolescence, the receipt of rewards has been shown to drive learning behaviors and suppress inappropriate actions (Lourenco and Casey, 2013). Rehabilitative efforts that capitalize on youth’s existing strengths and incentivize learning through rewards vs. punishments are neurodevelopmentally poised to succeed.

Funding more rehabilitative options for adjudicated youth may lead to less youth entering juvenile jails, detention centers, and adult prisons, diminishing the incidence of confinement-related stressors. It may also lead to greater cost-benefit outcomes. For example, multisystemic therapy provided for serious juvenile offenders resulted in $4,643 saved per youth in behavioral health claims (Dopp et al., 2018). While systemic barriers attributable to funding, existing policies, and regulations often curtail reform (Guckenburg et al., 2019), scientific advancements in neurodevelopment have already contributed to the improved treatment of youth. Since the landmark Supreme Court cases over a decade ago, further scientific advancements have been made addressing neurodevelopment in the context of adversity exposure, making the juvenile carceral system long overdue for the integration of neuroscientific understandings in the treatment of youth. In addition to improved rehabilitative outcomes and reintegration into society, the inclusion of this information may help shift the public’s perception of and long-term investment in these youth.

## Conclusion

Due to neuroplasticity, adolescents are susceptible to lasting neural alterations in response to environmental conditions, especially the harsh conditions of juvenile confinement; however, they may also be more amenable than adults toward redirection and rehabilitation. To capitalize on adolescents’ unique rehabilitative potential, the primary objective of juvenile justice reform should be to strengthen and support redirection and rehabilitative efforts that are developmentally appropriate for youth and reinforce individual existing strengths and contributions. Advancement toward a developmentally appropriate response to youth who come to the attention of law enforcement requires acknowledgment that existing conditions may often constitute a state-sanctioned form of adversity exposure and exacerbation, an argument that the neuroscientific, pediatric, and behavioral health care communities can advance in striving toward decarceration of youth.

## Data availability statement

The original contributions presented in the study are included in the article/supplementary material, further inquiries can be directed to the corresponding author/s.

## Author contributions

NO conceptualized and designed the study, drafted the initial manuscript, reviewed, and revised the manuscript. AG, EB, PC, and ES critically reviewed the manuscript for important intellectual content. All authors approved the final manuscript as submitted and agreed to be accountable for all aspects of the work.

## Abbreviations

JJS: Juvenile Justice System
UN: United Nations
US: United States
LGBTQ: lesbian, gay, bisexual, transgender, and questioning
PTSD: post-traumatic stress disorder
HPA: hypothalamic-pituitary-adrenal
TBI: traumatic brain injury
SES: socioeconomic status.

## References

[B1] Aalsma M. C. Lau K. S. L. Perkins A. J. Schwartz K. Tu W. Wiehe S. E. (2016). Mortality of youth offenders along a continuum of justice system involvement. *Am. J. Prev. Med.* 50 303–310. 10.1016/j.amepre.2015.08.030 26585053

[B2] Abram K. M. Azores-gococo N. M. Emanuel K. M. Aaby D. A. Welty L. J. Hershfield J. A. (2017). Sex and racial/ethnic differences in positive outcomes in delinquent youth after detention a 12-year longitudinal study. *JAMA Pediatr.* 3078 123–132. 10.1001/jamapediatrics.2016.3260 27992626 PMC5704941

[B3] Abrams L. S. Jordan S. P. Montero L. A. (2018). What is a juvenile? A cross-national comparison of youth justice systems. *Youth Justice* 18 1–20. 10.1177/1473225418779850

[B4] American Civil Liberties Union (2013). *Alone and afraid: Children held in solitary confinement and isolation in juvenile detention and correctional facilities.* New York, NY: ACLU.

[B5] Anderson V. R. Ouyang F. Tu W. Rosenman M. B. Wiehe S. E. Aalsma M. C. (2019). Medicaid coverage and continuity for juvenile justice-involved youth. *J. Correct. Health Care* 25 45–54. 10.1177/1078345818820043 30616497

[B6] Avishai-Eliner S. Yi S. J. Baram T. Z. (1996). Developmental profile of messenger RNA for the corticotropin-releasing hormone receptor in the rat limbic system. *Dev. Brain Res.* 91 159–163. 8852365 10.1016/0165-3806(95)00158-1 PMC3408243

[B7] Baglivio M. T. Epps N. (2014). The prevalence of adverse childhood experiences (ACE) in the lives of juvenile offenders. *J. Juv. Justice* 3 1–23.

[B8] Bailoo J. D. Murphy E. Boada-Saña M. Varholick J. A. Hintze S. Baussière C. (2018). Effects of cage enrichment on behavior, welfare and outcome variability in female mice. *Front. Behav. Neurosci.* 12:232. 10.3389/fnbeh.2018.00232 30416435 PMC6212514

[B9] Balsamo D. N. Poncin Y. B. (2016). Community-based alternatives to incarceration and assessment and community-based planning for probation/community-based alternative. *Child Adolesc. Psychiatr. Clin. North Am.* 25 123–128. 10.1016/j.chc.2015.08.005 26593124

[B10] Barnert E. S. (2020). COVID-19 and youth impacted by juvenile and adult criminal justice systems. *Pediatrics* 146:e20201299. 10.1542/peds.2020-1299 32709736 PMC7397728

[B11] Barnert E. S. Abrams L. S. Dudovitz R. Coker T. R. Bath E. Tesema L. (2019). What is the relationship between incarceration of children and adult health outcomes? *Acad. Pediatr.* 19 342–350. 10.1016/j.acap.2018.06.005 29935252 PMC6309510

[B12] Barnert E. S. Perry R. Morris R. E. (2016). Juvenile incarceration and health. *Acad. Pediatr.* 16 99–109. 10.1016/j.acap.2015.09.004 26548359

[B13] Bath K. G. Manzano-nieves G. Goodwill H. (2016). Hormones and behavior early life stress accelerates behavioral and neural maturation of the hippocampus in male mice. *Horm. Behav.* 82 64–71. 10.1016/j.yhbeh.2016.04.010 27155103 PMC5308418

[B14] Biggio F. Mostallino M. C. Talani G. Locci V. Mostallino R. Calandra G. (2019). Social enrichment reverses the isolation-induced deficits of neuronal plasticity in the hippocampus of male rats. *Neuropharmacology* 151 45–54. 10.1016/j.neuropharm.2019.03.030 30935859

[B15] Blakemore S. J. Burnett S. Dahl R. E. (2010). The role of puberty in the developing adolescent brain. *Hum. Brain Mapp.* 31 926–933. 10.1002/hbm.21052 20496383 PMC3410522

[B16] Bos M. G. N. Wierenga L. M. Blankenstein N. E. Schreuders E. Tamnes C. K. Crone E. A. (2018). Longitudinal structural brain development and externalizing behavior in adolescence. *J. Child Psychol. Psychiatry* 10 1061–1072. 10.1111/jcpp.12972 30255501 PMC6175471

[B17] Callaghan B. L. Sullivan R. M. Howell B. (2014). The international society for developmental psychobiology Sackler symposium: Early adversity and the maturation of emotion circuits — A cross-species analysis. *Dev. Psychobiol.* 56 1635–1650. 10.1002/dev.21260 25290865 PMC4831705

[B18] Champagne F. A. Meaney M. J. (2007). Transgenerational effects of social environment on variations in maternal care and behavioral response to novelty. *Behav. Neurosci.* 121 1353–1363. 10.1037/0735-7044.121.6.1353 18085888

[B19] Chapman J. F. Desai R. A. Falzer P. R. Borum R. (2006). Violence risk and race in a sample of youth in juvenile detention. The potential to reduce disproportionate minority confinement. *Youth Viol. Juv. Justice* 4 170–184. 10.1177/1541204006286316

[B20] Cowan C. S. M. Callaghan B. L. Kan J. M. Richardson R. (2016). The lasting impact of early-life adversity on individuals and their descendants: Potential mechanisms and hope for intervention. *Genes Brain Behav.* 15 155–168. 10.1111/gbb.12263 26482536

[B21] Department of Justice (2017). *Department of justice announces new reforms to strengthen the federal bureau of prisons. Press release number: 16-493.* Available online at: https://www.justice.gov/opa/pr/department-justice-announces-new-reforms-strengthen-federal-bureau-prisons/ (accessed October 24, 2017).

[B22] Dopp A. R. Coen A. S. Smith A. B. Reno J. Bernstein D. H. Kerns S. E. U. (2018). Economic impact of the statewide implementation of an evidence-based treatment: Multisystemic therapy in new Mexico. *Behav. Ther.* 49 551–566. 10.1016/j.beth.2017.12.003 29937257

[B23] Ewing-Cobbs L. Prasad M. Landry S. (1999). Inflicted traumatic brain injury: Relationship of developmental outcome to severity of injury. *Pediatr. Neurosurg.* 77030 251–258. 10.1159/000028872 10681680

[B24] Fagan A. A. Wright E. M. Pinchevsky G. M. (2014). The protective effects of neighborhood collective efficacy on adolescent substance use and violence following exposure to violence. *J. Youth Adolesc.* 43 1498–1512. 10.1007/s10964-013-0049-8 24170438 PMC4006326

[B25] Farahmand F. K. Duffy S. N. Tailor M. A. Dubois D. L. Lyon A. L. Grant K. E. (2012). Community-based mental health and behavioral programs for low-income urban youth: A meta-analytic review. database of abstracts of reviews and effects (DARE): Wuality-assessed reviews. *Clin. Psychol.* 19 195–215.

[B26] Farrer T. J. Frost R. B. Hedges D. W. (2013). Prevalence of traumatic brain injury in juvenile offenders: A meta-analysis. *Child Neuropsychol.* 19 225–234. 10.1080/09297049.2011.647901 22372420

[B27] Feld B. C. (1997). Abolish the juvenile court: Youthfulness, criminal responsibility, and sentencing policy. *J. Crim. Law Criminol.* 88 68–136.

[B28] Felitti V. J. Anda R. F. Nordenberg D. Williamson D. F. Spitz A. M. Edwards V. (1998). Household dysfunction to many of the leading causes of death in adults: The adverse childhood experiences (ACE) study. *Am. J. Prev. Med.* 14 245–258. 9635069 10.1016/s0749-3797(98)00017-8

[B29] Fritz J. de Graaff A. M. Caisley H. van Harmelen A.-L. Wilkinson P. O. (2018). A systematic review of amenable resilience factors that moderate and/or mediate the relationship between childhood adversity and mental health in young people. *Front. Psychiatry* 9:230. 10.3389/fpsyt.2018.00230 29971021 PMC6018532

[B30] Guckenburg S. Stern A. Sutherland H. Lopez G. Petrosino A. (2019). *Juvenile detention alternatives initiative scale-up: Study of four states.* San Francisco, CA: WestEd.

[B31] Hanson J. L. Hariri A. R. Williamson D. E. (2015). Blunted ventral striatum development in adolescence reflects emotional neglect and predicts depressive symptoms. *Biol. Psychiatry* 78 598–605. 10.1016/j.biopsych.2015.05.010 26092778 PMC4593720

[B32] Herting M. M. Sowell E. R. (2017). Puberty and structural brain development in humans. *Front. Neuroendocrinol.* 44:122–137. 10.1016/j.yfrne.2016.12.003 28007528 PMC5612369

[B33] Justice Policy Institute (2015). *Factsheet: The tip of the iceberg: What taxpayers pay to incarcerate youth.* Washington, DC: Justice Policy Institute.

[B34] Kuhlman K. R. Chiang J. J. Horn S. Bower J. E. (2018). Developmental psychoneuroendocrine and psychoneuroimmune pathways from childhood adversity to disease. *Neurosci. Biobehav. Rev.* 80 166–184. 10.1016/j.neubiorev.2017.05.020.Developmental PMC5705276 28577879

[B35] Lansing A. E. Virk A. Notestine R. Plante W. Y. Fennema-notestine C. (2016). Psychiatry research: Neuroimaging cumulative trauma, adversity and grief symptoms associated with fronto-temporal regions in life-course persistent delinquent boys. *Psychiatry Res. Neuroimaging* 254 92–102. 10.1016/j.pscychresns.2016.06.007 27388804 PMC4992608

[B36] Lipsey M. W. (2009). The primary factors that characterize effective interventions with juvenile offenders: A meta-analytic overview. *Victims Offend.* 4 124–147. 10.1080/15564880802612573

[B37] Logan-Greene P. Kim B. K. E. Nurius P. S. (2016). Childhood adversity among court-involved youth: Heterogeneous needs for prevention and treatment. *J. Juv. Justice* 5 68–84. 31404461 PMC6688767

[B38] Lourenco F. Casey B. J. (2013). Adjusting behavior to changing environmental demands with development. *Neurosci. Biobehav. Rev.* 37(9 Pt B) 2233–2242. 10.1016/j.neubiorev.2013.03.003 23518271 PMC3751996

[B39] Lupien S. J. Mcewen B. S. Gunnar M. R. Heim C. (2009). Effects of stress throughout the lifespan on the brain, behaviour and cognition. *Nature* 10 434–445. 10.1038/nrn2639 19401723

[B40] Lynch M. Esthappan S. Astone N. M. Collazos J. Lipman M. (2018). *Arches transformative mentoring program.* New York, NY: Urban Institute.

[B41] McCarthy P. Schiraldi V. Shark M. (2016). *New thinking in community corrections.* Washington, DC: U.S. Department of Justice.

[B42] McKinlay A. (2014). Long-term outcomes of traumatic brain injury in early childhood. *Austral. Psychol. Soc.* 49 323–327. 10.1111/ap.12084

[B43] McLaughlin K. A. Weissman D. Bitrán D. (2019). Childhood adversity and neural development: A systematic review. *Annu. Rev. Dev. Psychol.* 1 277–312. 10.1146/annurev-devpsych-121318-084950 32455344 PMC7243625

[B44] Mendel R. A. (2011). *No place for kids: The case for reducing juvenile incarceration.* Baltimore, MD: Annie E. Casey Foundation.

[B45] Mendel R. A. (2015). *Maltreatment of youth in U.S. juvenile corrections facilities: An update.* Baltimore, MD: Annie E. Casey Foundation.

[B46] Mendle J. Beltz A. M. Carter R. (2019). Understanding puberty and its measurement: Ideas for research in a new generation. *J. Res. Adolesc.* 29 82–95. 10.1111/jora.12371 30869839

[B47] Mota C. P. Costa M. Matos P. M. (2016). Resilience and deviant behavior among institutionalized adolescents: The relationship with significant adults. *Child Adolesc. Soc. Work* 33 313–325. 10.1007/s10560-015-0429-x

[B48] Motley R. Sewell W. Chen Y. C. (2017). Community violence exposure and risk-taking behaviors among black emerging adults: A systematic review. *J. Commun. Health* 42 1069–1078. 10.1007/s10900-017-0353-4 28421427 PMC5647207

[B49] Nettle D. Andrews C. Reichert S. Bed T. Kolenda C. Parker C. (2017). Early-life adversity accelerates cellular ageing and affects adult inflammation: Experimental evidence from the European starling. *Sci. Rep.* 7:40794. 10.1038/srep40794 28094324 PMC5240102

[B50] Office of Juvenile Justice and Delinquency Juvenile Arrests (2021). *Juvenile justice statistics.* Available online at: https://ojjdp.ojp.gov/publications/juvenile-arrests-2019.pdf (accessed June 15, 2021).

[B51] Owen M. C. Wallace S. B. (2020). Advocacy and collaborative health care for justice-involved youth. *Pediatrics* 146:e20201755. 10.1542/peds.2020-1755 32376728

[B52] Padgaonkar N. T. Baker A. E. Dapretto M. Galván A. Frick P. J. Steinberg L. (2020). Exploring disproportionate minority contact in the juvenile justice system over the year following first arrest. *J. Res. Adolesc.* 31, 317–334. 10.1111/jora.12599 33280192 PMC8127356

[B53] Ramnitz M. S. Lodish M. B. (2013). Racial disparities in pubertal development. *Semin. Reprod. Med.* 31 333–339. 10.1055/s-0033-1348891 23934693

[B54] Sinclair D. Purves-Tyson T. D. Allen K. M. Weickert C. S. (2014). Impacts of stress and sex hormones on dopamine neurotransmission in the adolescent brain. *Psychopharmacology* 231 1581–1599. 10.1007/s00213-013-3415-z 24481565 PMC3967083

[B55] Society for Adolescent Health and Medicine (2016). International youth justice systems: Promoting youth development and alternative approaches: A position paper of the society for adolescent health and medicine society for adolescent health and medicine. *J. Adolesc. Health* 59 482–486. 10.1016/j.jadohealth.2016.08.003 27664466

[B56] Spinney E. Cohen M. Feyerherm W. Stephenson R. Yeide M. Shreve T. (2018). Disproportionate minority contact in the U.S. juvenile justice system: A review of the DMC literature, 2001-2014, part I. *J. Crime Justice* 41 573–595. 10.1080/0735648X.2018.1516155

[B57] Steinberg L. (2013). The influence of neuroscience on US Supreme Court decisions about adolescents’ criminal culpability. *Nat. Publ. Group* 14 513–518. 10.1038/nrn3509 23756633

[B58] Sumner J. A. Colich N. L. Uddin M. Armstrong D. Mclaughlin K. A. (2019). Early experiences of threat, but not deprivation, are associated with accelerated biological aging in children and adolescents. *Biol. Psychiatry* 85 268–278. 10.1016/j.biopsych.2018.09.008 30391001 PMC6326868

[B59] Teicher M. H. Samson J. A. (2016). Annual research review: Enduring neurobiological effects of childhood abuse and neglect. *J. Child Psychol. Psychiatry Allied Discipl.* 57 241–266. 10.1111/jcpp.12507 26831814 PMC4760853

[B60] Teicher M. H. Anderson C. M. Polcari A. (2012). Childhood maltreatment is associated with reduced volume in the hippocampal subfields CA3, dentate gyrus, and subiculum. *Proc. Natl. Acad. Sci. U.S.A.* 109 E563–E572. 10.1073/pnas.1115396109 22331913 PMC3295326

[B61] The Annie E. Casey Foundation (2017). *Juvenile detention alternatives initiative at 25: Insights from the annual results reports.* Baltimore, MD: The Annie E. Casey Foundation.

[B62] Tottenham N. Galván A. (2016). Neuroscience and biobehavioral reviews stress and the adolescent brain amygdala-prefrontal cortex circuitry and ventral striatum as developmental targets. *Neurosci. Biobehav. Rev.* 70 217–227. 10.1016/j.neubiorev.2016.07.030 27473936 PMC5074883

[B63] Williams D. R. Collins C. (2001). Racial residential segregation: A fundamental cause of racial disparities in health. *Public Health Rep.* 116 404–416. 10.1093/phr/116.5.404 12042604 PMC1497358

[B64] Wilson H. W. Stover C. S. Berkowitz S. J. (2009). Research review: The relationship between childhood violence exposure and juvenile antisocial behavior: A meta-analytic review. *J. Child Psychol. Psychiatry* 7 769–779. 10.1111/j.1469-7610.2008.01974.x 19017367

[B65] Winiarski D. A. Schechter J. C. Brennan P. A. Foster S. L. Cunningham P. B. Whitmore E. A. (2017). Adolescent physiological and behavioral patterns of emotion dysregulation predict multisystemic therapy response. *J. Emot. Behav. Disord.* 25 131–142. 10.1177/1063426616638315 28867925 PMC5580832

